# Intracranial Traumatic Hematoma Detection in Children Using a Portable Near-infrared Spectroscopy Device

**DOI:** 10.5811/westjem.2020.11.47251

**Published:** 2021-03-24

**Authors:** Matthew P. Kirschen, Sage R. Myers, Mark I. Neuman, Joseph A. Grubenhoff, Rebekah Mannix, Nicholas Stence, Edward Yang, Ashley L. Woodford, Tyson Rogers, Anna Nordell, Arastoo Vossough, Mark R. Zonfrillo

**Affiliations:** * Children’s Hospital of Philadelphia, Department of Anesthesiology and Critical Care Medicine, Philadelphia, Pennsylvania; † Children’s Hospital of Philadelphia, Division of Neurology, Philadelphia, Pennsylvania; ‡ Children’s Hospital of Philadelphia, Department of Pediatrics, Philadelphia, Pennsylvania; § Children’s Hospital of Philadelphia, Division of Emergency Medicine, Philadelphia, Pennsylvania; ¶ Boston Children’s Hospital, Division of Emergency Medicine, Department of Pediatrics, Boston, Massachusetts; || University of Colorado, Department of Pediatrics, Aurora, Colorado; # Children’s Hospital Colorado, Department of Radiology, Aurora, Colorado; ** Boston Children’s Hospital, Department of Radiology, Boston, Massachusetts; †† Cooper Medical School of Rowan University, Camden, New Jersey; ‡‡ North American Science Associates Inc., Minneapolis, Minnesota; §§ Children’s Hospital of Philadelphia, Department of Radiology, Philadelphia, Pennsylvania; ¶¶ Alpert Medical School of Brown University, Departments of Emergency Medicine and Pediatrics, Providence, Rhode Island

## Abstract

**Introduction:**

We sought to validate a handheld, near-infrared spectroscopy (NIRS) device for detecting intracranial hematomas in children with head injury.

**Methods:**

Eligible patients were those <18 years old who were admitted to the emergency department at three academic children’s hospitals with head trauma and who received a clinically indicated head computed tomography (HCT). Measurements were obtained by a blinded operator in bilateral frontal, temporal, parietal, and occipital regions. Qualifying hematomas were a priori determined to be within the brain scanner’s detection limits of >3.5 milliliters in volume and <2.5 centimeters from the surface of the brain. The device’s measurements were positive if the difference in optical density between hemispheres was >0.2 on three successive scans. We calculated diagnostic performance measures with corresponding exact two-sided 95% Clopper-Pearson confidence intervals (CI). Hypothesis test evaluated whether predictive performance exceeded chance agreement (predictive Youden’s index > 0).

**Results:**

A total of 464 patients were enrolled and 344 met inclusion for primary data analysis: 10.5% (36/344) had evidence of a hematoma on HCT, and 4.7% (16/344) had qualifying hematomas. The handheld brain scanner demonstrated a sensitivity of 58.3% (21/36) and specificity of 67.9% (209/308) for hematomas of any size. For qualifying hematomas the scanner was designed to detect, sensitivity was 81% (13/16) and specificity was 67.4% (221/328). Predictive performance exceeded chance agreement with a predictive Youden’s index of 0.11 (95% CI, 0.10 – 0.15; P < 0.001) for all hematomas, and 0.09 (95% CI, 0.08 – 0.12; P < 0.001) for qualifying hematomas.

**Conclusion:**

The handheld brain scanner can non-invasively detect a subset of intracranial hematomas in children and may serve an adjunctive role to head-injury neuroimaging decision rules that predict the risk of clinically significant intracranial pathology after head trauma.

## INTRODUCTION

Nearly 760,000 children and adolescents less than 18 years old with head trauma undergo evaluation in United States (US) emergency departments (ED) annually.^1^ While there are multiple decision rules that predict the risk of a significant intracranial injury in children with head trauma, a substantial proportion of children classified as “not low” risk may require an extended period of observation or neuroimaging to exclude the presence of an intracranial hematoma that may require neurosurgical intervention.^2^ An expanding hematoma can lead to significant neurological morbidity or death due to brainstem compression or further ischemic injury. A computed tomography scan of the head (HCT) is the clinical standard for emergent identification and localization of acute intracranial hematomas. However, the ionizing radiation increases the risk of developing malignancies.^3^ Notably, 26% of children evaluated for mild head injury will undergo a HCT, thus exposing a large population of children each year to ionizing radiation.^4^

Near-infrared spectroscopy (NIRS) is a non-invasive, radiation-sparing technology that measures the near- infrared light absorption of hemoglobin within the brain and may be useful as an adjunctive modality for early identification of intracranial hematomas in patients with head trauma.^5^–^7^ Extravascular hemoglobin absorbs more near-infrared light (usually 10-fold) than intravascular hemoglobin, enabling NIRS devices to detect differential absorption between intracranial hematomas and uninjured brain. A handheld NIRS detection system, the Infrascanner Model 2000 (InfraScan, Inc., Philadelphia, PA) has shown 90% sensitivity and specificity for detecting intracranial hematomas in adult patients suffering from head trauma.^8^,^9^

Given the fixed size of the device and the differences between adult and pediatric cranial anatomy (eg, cranial bone thickness and composition, presence of cranial sutures, and brain volume and composition) and head trauma mechanics, it was unknown whether the device would display similar performance characteristics in children. Previous pediatric studies conducted with an earlier model of the device each detected only a few hematomas, and used different patient populations, scanning protocols, and incomplete blinding.^8^–^14^ This study is important because we designed it to overcome those limitations by using the newer model scanner in a multicenter approach with blinded operators and independent neuroradiological review of HCTs to more precisely determine the device’s performance characteristics in children with head injury.

We aimed to validate the Infrascanner Model 2000 in children of all ages with known or suspected head injury compared to HCT as the clinical standard. We hypothesized that, compared to HCT, the device would have a sensitivity non-inferior to 90% (the sensitivity found in adult trials) to detect intracranial hematomas that are within the detection limit of the device.

**Population Health Research Capsule**

What do we already know about this issue?

*An expanding intracranial hematoma after traumatic brain injury can lead to significant neurological morbidity or death due to brainstem compression or ischemia.*

What was the research question?

*How does Infrascanner compare to head CT for detecting intracranial hematomas in children with head injury?*

What was the major finding of the study?

*The Infrascanner demonstrated a sensitivity of 81% and specificity of 67% for detecting qualifying hematomas.*

How does this improve population health?

*The Infrascanner device may serve an adjunctive role to head injury imaging decision rules that predict risk of intracranial pathology after pediatric head trauma.*

## METHODS

### Study Design and Setting

We conducted an observational device validation study between June 2014–September 2018 in the EDs of three large, urban, quaternary care academic medical centers: Children’s Hospital of Philadelphia, Boston Children’s Hospital, Children’s Hospital Colorado. Enrollment at the latter two centers began in April and June 2016, respectively. The protocol was approved by the institutional review board at each institution. The study was registered on ClinicalTrials.gov with the identifier NCT02149082. The Infrascanner Model 2000 has 510(k) clearance (K120949) from the US Food and Drug Administration (FDA) as a Class II device for individuals 18 years of age and older.

### Selection of Participants

Eligible participants were individuals less than 18 years old presenting to the ED with known or suspected head trauma who received a clinically indicated HCT. This was a convenience sample as enrollment occurred only when research team members were available for enrollment. If a research team member was not available for a shift, the census was screened the following shift and eligible patients were recorded as not enrolled due to lack of staff availability. Patients who received an initial HCT after trauma or HCT performed for a clinical change (eg, seizure, headache, emesis, focal neurological deficit) were eligible. The HCTs were required to be performed within 12 hours of trauma or clinical change. Hemoglobin in an intracranial hematoma begins transitioning into methemoglobin after about 12 hours, after which it is not detectable by the NIRS sensor. Patients were excluded if they had the following: extensive scalp injury including lacerations, avulsions, or abrasions that prevented proper application of the device to the patient’s head or prevented placement of the device in the specified locations; or they had a history of a neurosurgical procedure (eg, decompressive craniectomy) with residual bone flap.

Since intracranial hematomas are dynamic and evolve over time, it was important to minimize the time between the scanner exam and HCT. The scanner measurement had to be completed within six hours before or after the HCT. This was increased from 40 minutes during the study due to a larger than expected number of patients at the lead site who had HCTs performed at referring hospitals prior to transfer and were excluded due to duration from time of HCT. Parents or legal guardians were required to provide verbal consent in person or via telephone. Patient assent was not required, but patients who dissented were not enrolled.

Research coordinators and assistants were operators of the handheld brain scanner at each site. Operators at the original site underwent training by representatives from InfraScan and the investigators. Operators attended practice sessions followed by a proficiency assessment by an investigator. Operators at the other sites were trained by the principal investigator and lead research coordinator from the lead site. Operators who joined the study after initiation were trained by investigators and lead coordinators at each site. Operators were instructed to perform several measurements per month to maintain proficiency, and they underwent refresher and practice sessions if they had not enrolled a patient in several weeks. These procedures were instituted after pilot testing indicated frequent use of the device was necessary to ensure proper use and strict adherence to the standardized protocol. Operators were blinded to HCT results. After operator training at the lead site, there was no involvement by the company.

### Measurements

**Figure 1:**
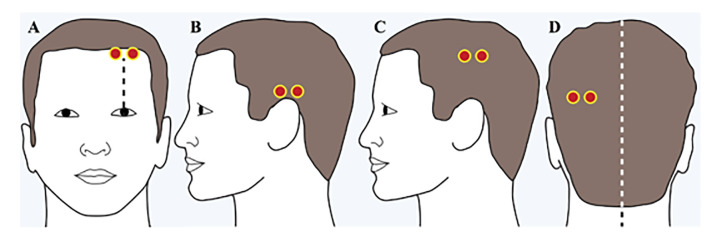
Standardized measurement locations for the handheld brain scanner device.

The handheld brain scanner was placed successively in the left and right frontal, temporal, parietal, and occipital regions of the head, and the absorbance of light was recorded (Figure 1).

We calculated the difference in optical density (ΔOD) between the right and left hemisphere in each of the four regions on a pairwise basis using the equation ΔOD = log_10_ (I_N_/I_H_) where I_N_ is the intensity of the reflected light on the presumed normal side, and I_H_ is the intensity of the reflected light on the presumed abnormal side.^9^ A predefined ΔOD threshold of >0.2 was determined to be positive for a hematoma based on a pilot study of patients with hematomas and healthy controls and set to maximize sensitivity and specificity accounting for inter-operator reliability, variability due to accidental hair compression, and distribution of the NIRS signal within hematomas.^5^ In each brain region where the ΔOD was >0.2 the operator repeated the exam in the same region two additional times to confirm the positive measurement. This procedure was designed to reduce the likelihood of a false positive measurement due to an impinged hair under the device or asymmetrical placement. If a ΔOD was ≤0.2 at any measurement (independent of whether a prior measurement was positive in the same region), the region was noted as negative and the operator moved to the next successive region. Operators could use the device in either a “guided mode” where step-by-step instructions were provided or an “independent mode.”

Operators noted areas of scalp hematomas, ecchymoses, abrasions, and small lacerations, and were instructed to reposition the device slightly to avoid these areas to limit false positive measurements. The occipital region was deferred if the patient had a cervical immobilization collar in place. Operators recorded skin color as light/white, olive/brown, or black, and hair color as scant, blond, red, brown, or black since darker hair or skin color may alter light absorption and affect the NIRS measurements.

Operators recorded demographic information about each patient including clinical data relating to head trauma. Glasgow Coma Scale scores were obtained from the treating clinician. Health record review was conducted for each enrolled patient to determine whether the patient had a clinically important traumatic brain injury (TBI) defined as TBI-related death, neurosurgical intervention, intubation of more than 24 hours, or hospital admission of two nights or more for the TBI in association with TBI on HCT.^15^

The HCTs were interpreted by pediatric neuroradiologists at each site and blinded to the handheld device result and the clinical radiology report. Intracranial hematomas were characterized by location (ie, epidural, subdural, intraparenchymal, or subarachnoid), volume, and distance from the cortical surface. Hematoma volume was calculated using standardized methods (primarily ABC/2) based on location by either a neuroradiologist or a trained research coordinator.^16^–^19^ All HCTs with hematomas were reviewed at Children’s Hospital of Philadelphia by a blinded, independent pediatric neuroradiologist to confirm hematoma characteristics.

### Outcomes Measures

Patients were considered evaluable if device measurements were assessed in three or four symmetrical brain regions. This was to account primarily for deferring the occipital region due to cervical immobilization collars. A qualifying hematoma was defined to be a hematoma within the predefined detection limit of the Infrascanner device if it was >3.5 milliliters (mL) in volume and <2.5 centimeters (cm) from the surface of the brain.^5^,^8^ A positive Infrascanner measurement required the hematoma be confirmed on three successive assessments.

### Data Analysis

We summarized continuous measures using mean and standard deviation or median and interquartile range and categorical measures as counts and percentages. Diagnostic performance measures (sensitivity, specificity, negative predictive value [NPV], positive predictive value [PPV]) included a corresponding exact two-sided 95% Clopper-Pearson confidence interval (CI).^20^ The Youden’s index (sensitivity plus specificity minus 1) and an analogue based on predictive value metrics (NPV + PPV − 1) were used to assess the degree to which the performance of the device exceeded the performance that could be explained by chance alone. We calculated two-sided CIs for these Youden statistics by applying the Wilson score interval method for a binomial proportion.^21^ Consistency of performance across subgroups with different baseline characteristics was assessed by stratification, with a chi-square test to compare performance across strata. Pre-specified subgroups included age, hematoma location, hematoma volume, and presence of extracranial or scalp soft-tissue hematomas. Each operator’s diagnostic performance was compared to other operators with a chi-square test with a Bonferroni *P*-value adjustment for multiple comparisons.

The hypothesis that sensitivity was non-inferior to 90% with a 10% margin, 80% power, and 5% type 1 error yielded a calculated sample size of 383 enrollments to identify 82 hematomas within detection limits. During the study, it was recognized that this hypothesis test was not viable due to low prevalence of intracranial hematomas and corresponding low power. After consultation with the company and the FDA, an alternative hypothesis was planned prior to unblinded data analysis. This hypothesis test assesses the predictive analogue of Youden’s index (NPV + PPV − 1) for performance better than expected by chance with power >90% and 2.5% type 1 error with the available sample size of 344. The predictive Youden’s index summarizes the performance of a diagnostic test with values ranging from −1 to 1. Zero denotes a test that whose diagnoses are correct at the rate expected by chance (test is useless), −1 indicates all diagnoses are incorrect, and 1 indicates that all diagnoses are correct (test is perfect).

## RESULTS

### Characteristics of Study Subjects

**Figure 2:**
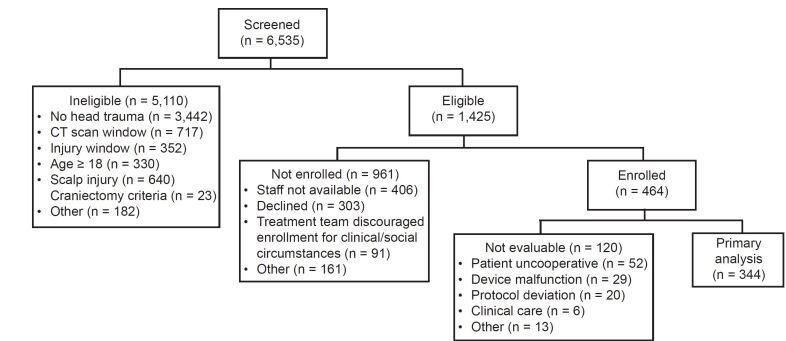
Flow chart for patient enrollment. *CT*, computed tomography.

We assessed a total of 6535 patients for inclusion: 1425 were eligible, 464 were enrolled, and 344 met inclusion for primary data analysis (Table 1) by having handheld scanner measurements correctly completed on three or four brain regions (Figure 2). Site enrollment was as follows: 54% (186/344) of patients were enrolled at Children’s Hospital of Philadelphia, 19% (66/344) at Boston Children’s Hospital, and 27% (92/344) at Children’s Hospital Colorado.

### Main Results

Overall, 10.5% (36/344) of patients had neuroradiological evidence of a hematoma on HCT, and 4.7% (16/344) had hematomas that were within the detection limit of the device. Of these 16 evaluable hematomas, nine were epidural, four subdural, and three intraparenchymal, with an average volume of 19.0 mL (range 4.6 – 53.0 mL).

For all hematomas, the Infrascanner demonstrated sensitivity of 58% (21/36), specificity of 68% (209/308), PPV of 18% (21/120), and NPV of 93% (209/224) (Table 2). For hematomas within the device’s detection limits, the Infrascanner demonstrated sensitivity of 81% (13/16), specificity of 67% (221/328), PPV of 11% (13/120), and NPV of 99% (221/224). Both Youden’s index and its predictive analogue were statistically significantly greater than zero for all hematomas and those within the device’s detection limits (*P*<0.001 for all; Table 2).

Diagnostic performance was independent of age (divided by quartile), hair/skin color, and race. Diagnostic performance was also independent of whether three (43% 149/344) or four (57% 195/344) brain regions were assessed. Of the 149 patients with three lobes measured, the deferred lobe was occipital for 117 (79%) of patients, primarily due to presence of a cervical immobilization collar. Diagnostic performance was also comparable across the three sites, between the device’s “independent” and “guided” modes, and between patients with and without documented scalp hematomas (Supplementary Table 1).

There were 24 trained operators at Children’s Hospital of Philadelphia, 11 at Boston Children’s Hospital, and 10 at Children’s Hospital Colorado, each completing an average of 10.3 (range 1–61) assessments. One operator was found to be an outlier with regard to specificity (Supplementary Figure 1), with a specificity of 42.9% (15/35) vs 71.1% (194/273) for the remaining operators (*P* = 0.0008). The operator was determined to be an outlier using a Bonferroni-adjusted *P*-value threshold of 0.001 (0.05/45) to account for the 45 different operators evaluated. After excluding this operator, there was no significant association between false positive rate and operator experience (ie, number of scans performed stratified by quartile; *P*-value = 0.14).

The median time between the HCT and the handheld scanner assessment was 53 [IQR 25–150] minutes. We evaluated whether device performance was associated with the time interval between the HCT and the handheld device assessment since prior Infrascanner studies used a maximal interval between the HCT and device assessment of 40 minutes,^8^ and this study initially had a limit of 40 minutes prior to 2016 when we lengthened the maximum to six hours to address enrollment issues. Diagnostic performance was not associated with interval in a logistic regression model (*P* = 0.24 for sensitivity, *P* = 0.29 for specificity).

We were unable to determine the average time to complete an Infrascanner assessment due to interruptions for clinical care and patient cooperation, although operators reported that the assessment was typically completed in 3–5 minutes. There were no adverse events reported. A total of 127 (26%) of the enrolled patients were not evaluable (Figure 2). Infrascanner assessments were terminated early due to the patient being uncooperative (43%, 52/120), device malfunction (24%, 29/120), clinical care (5%, 6/120), protocol deviation (17%, 20/120); or other reasons (11%, 13/120).

**Figure 3:**
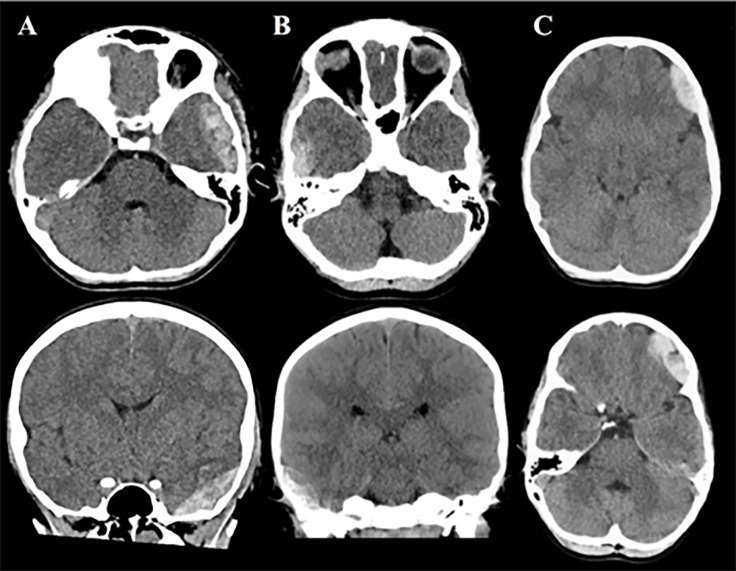
Computed tomography images of three hematomas not detected by the handheld brain scanner.

The handheld brain scanner failed to detect three hematomas that were within detection limits (ie, false negatives). All three were epidural hematomas (Figure 3). One patient had four lobes assessed and the other two patients had three lobes assessed. The deferred lobe was occipital for both patients, and neither hematoma was in the occipital region. Two patients had a clinically important TBI and required hospital admission for two or more nights (Table 3). The third patient did not meet criteria for a clinically important TBI. None of these patients required neurosurgical intervention.

The overall incidence of clinically important TBI amongst all evaluable patients was 4.9% (17/344), and 47.2% (17/36) in patients with an intracranial hematoma (Table 3). All evaluable patients with a clinically important TBI required hospital admission for two or more nights. Four of these patients also required neurosurgical intervention. All four had intracranial hematomas detected by the handheld brain scanner. Two patients with clinically important TBIs had hematomas that were outside the detection limits of the Infrascanner and were not detected by the device. These patients required hospital admission for two or more nights, but did not require intubation for more than 24 hours or neurosurgical intervention. There were no patient deaths.

Of the 120 patients who were enrolled and not evaluable, 14 (12%) patients had a hematoma on HCT. Three of these hematomas were within the detection limits of the device and one required neurosurgical intervention. Of the 11 patients who had hematomas outside the detection limit of the device, six patients had clinically significant TBIs. One patient required neurosurgical intervention and the other patients required hospital admission for two or more nights. Of the 106 patients who were enrolled and not evaluable and did not have a hematoma on HCT, five required hospital admission for two or more nights. As a worst-case sensitivity analysis, we computed the sensitivity if the 120 patients where the Infrascanner assessment was either not done (41/120) or was done but either the study was incomplete or there was a protocol deviation (79/120). Six of the 14 hematomas were detected by the device, yielding a worst-case sensitivity of 54% (27/50).

## DISCUSSION

We demonstrated in this multicenter pediatric device validation study that the Infrascanner handheld NIRS detection system had an NPV of 98.7% and a sensitivity of 81% compared to HCT for detecting intracranial hematomas within the detection limit of the device. The device’s specificity was 71.1% for any hematoma after accounting for the operator outlier. These results extend the growing body of literature evaluating the utility of this device for non-invasively detecting traumatic intracranial hematomas in children.^8^,^9^

Our sensitivity of 81% was comparable to what has been reported in the pediatric literature (85–100%).^9^ The undetected hematomas in our study likely resulted from a discrepancy between the location of the intracranial hematoma and the standardized Infrascanner probe positions on the scalp. The missed temporal hematomas (Figure 3 A and B) were likely inferior to the placement of the device (Figure 1). The placement of the scanner for the missed frontal bleed (Figure 3 C) was likely superior to hematoma location, partially due to ecchymoses around the patient’s ipsilateral orbit. While repositioning the device slightly to avoid areas of scalp injury or obvious hematomas is permissible per the standardized protocol, it may have contributed to the missed bleed and lower sensitivity. A Turkish study that evaluated 161 pediatrics patients found the device’s sensitivity to be 85.7%, although details about the location of missed hematomas and device placement were not provided.^10^

Our study also found a specificity of 71% after accounting for the operator outlier, which was near the low end of what has previously been reported in the pediatric literature (65–100%).^9^ One prior pediatric study reported a specificity of 65%, although in this study the lower specificity may have been impacted by the fact that operators were not required to confirm positive Infrascanner measurement three times.^10^ We used 45 operators across three sites for this study with a majority of the operators being trained by investigators and study coordinators. This contrasts prior pediatric Infrascanner studies that used only a few operators who were all trained by the company. After excluding one poor-performing operator, we found no association between operator experience and false positive rate. Finally, unmeasured patient- and operator-related factors may have contributed to the lower specificity in our study.

It’s worth noting that the detection limit of the device is for hematomas 3.5 mL in volume and 2.5 cm from the surface of the brain, which was determined from adult and phantom data.^22^ It is conceivable that the same size hematoma may be of greater clinical significance in a child than in an adult due to the fact that it will occupy a proportionally larger volume in the intracranial vault. A hematoma volume of 3.5 mL is approximately 1% of the total brain volume at birth and less than 1% of total brain volume for older children.^23^ One study found that a hematoma that was 2–4% of total brain volume yielded an elevated risk of moderate disability at three months.^24^ Hematomas ≤ 2% of total brain volume were not associated with severe disability or death. Therefore, it is likely that a hematoma detection size limit of 3.5 mL (~1% total brain volume) is sufficiently small as to be of limited clinical importance across all pediatric ages. Additionally, there is a lack of pediatric data and consensus regarding the association between the size of traumatic hematomas, clinical outcomes, and indications for surgical intervention.^25^–27

The device is unable to precisely determine the location (eg, subdural vs epidural) and volume extent of intracranial hematomas. Since Infrascanner measurements rely on comparing light absorption in contralateral brain regions, bilateral hematomas may be difficult to detect, and did not occur in any patients in this study. They are uncommon in clinical practice, and occur mostly in the setting of abusive head injury. The device is unable to detect infratentorial or brainstem hematomas with the current standardized protocol. Lastly, the device requires highly trained operators who maintain proficiency in standardized probe positioning, managing device error messages, and meticulous positioning of the device to avoid hair, foreign bodies, and scalp hematomas while applying the optimal pressure against to scalp to yield reliable measurements and limit patient discomfort. Design improvements that will render the device less operator dependent and reduce the need for training/retraining in future generations of the technology are recommended. These improvements have the potential to reduce the false positive rate preventing unnecessary HCT.

Given the high NPV of the Infrascanner device for detecting intracranial hematomas, it can serve an adjunctive role to decision rules that predict the risk of a significant intracranial injury in children with head trauma. For those children in a non-low risk category by prediction rule application, a negative Infrascanner assessment may obviate the need for neuroimaging or a prolonged period of observation. Our study provides preliminary evidence that studies of a larger cohort of children with head trauma, including more patients with variable hematoma sizes and locations, may help determine whether the Infrascanner’s diagnostic performance can be further improved by tailoring the ΔOD threshold of >0.2 or the standardized probe positions for children. It may also be beneficial to explore the role of this radiation-sparing technology in comparison to biochemical markers of traumatic brain injury.

## LIMITATIONS

Our study had several limitations. The main limitation was that the study was unable to enroll a sufficient number of subjects with intracranial hematomas to perform the planned hypothesis test of sensitivity. A total of 82 intracranial hematomas was required to yield 80% power. The final count of 16 hematomas within the detection limits of the device was low enough that power was 18.5% and only a sensitivity of 100% (16/16) would have resulted in passing the non-inferiority hypothesis test of sensitivity. As explained previously, an alternative hypothesis test of the predictive Youden’s index was developed prior to data unblinding. Whereas sensitivity only uses data from patients with intracranial hematomas, the predictive Youden’s index is a more comprehensive measure that assesses performance using data from all evaluable patients. Although the study did not reach the original planned sample size, the number of evaluable patients (n = 344) was sufficient to provide >90% power across a range of prevalence rates.

The reasons the study did not identify the planned 82 patients with intracranial hematomas were multifactorial and included that patients with severe TBIs who had the greatest likelihood of having intracranial hematomas required acute resuscitation, emergent neuroimaging, and transfer from the ED to either the operating room or the intensive care unit for further care and were not enrolled, although we do not have the exact number of patients where this occurred. Research staff did not have sufficient time or access to the patient to perform the Infrascanner assessment, and it was not feasible to obtain informed consent given the clinical circumstances and competing priorities. We categorized reasons why patients were ineligible on screening and why eligible patients were not enrolled. Four percent and seventeen percent of these patients, respectively, were categorized as “other” and we are unable to further determine the rationale. Twenty-six percent of enrolled patients were not evaluable.

Additionally, there was non-uniform training of study team members, as some were trained by representatives from the company and others by the lead investigator and research coordinator. Overall, fewer patients than expected presented with TBI and patients could only be enrolled when trained Infrascanner operators were available. Given the smaller than expected number of hematomas within the detection limit of the device, we did not have enough patients with intracranial hematomas to comprehensively evaluate the device’s performance by hematoma location or size, or the impact of distance from the surface of the brain for intraparenchymal hematomas.

## CONCLUSION

In summary, this non-invasive, radiation-sparing, NIRS-based technology may serve as an adjunct to current pediatric head injury neuroimaging decision rules for identifying intracranial hematomas in the ED setting. Further investigation to determine optimal training paradigms and the Infrascanner’s clinical impact, including subgroups of patients for whom its application can alter current imaging or observation patterns, is warranted.

## Supplementary Information





## Figures and Tables

**Table 1:** Patient and clinical characteristics.

	Evaluable patients (N = 344)	Any hematoma (N = 36)	Hematoma within the detection limit of the infrascanner (N = 16)
Age, mean [IQR]	9.5 [5.0, 13.8]	9.7 [4.3, 12.9]	10.4 [4.1, 12.9]
Male gender	225 (65%)	19 (53%)	9 (56%)
Race*			
Caucasian	168 (50%)	22 (67%)	9 (64%)
Black	120 (36%)	5 (15%)	2 (14%)
Asian	18 (5%)	1 (3%)	0 (0%)
Other	30 (9%)	5 (15%)	3 (21%)
Hispanic ethnicity*	54 (17%)	7 (22%)	5 (39%)
Skin color			
Light/white	161 (47%)	24 (67%)	10 (63%)
Black	106 (31%)	4 (11%)	1 (6%)
Olive/brown	77 (22%)	8 (22%)	5 (31%)
Hair color			
Black	143 (42%)	7 (20%)	4 (27%)
Brown	99 (29%)	14 (40%)	9 (60%)
Blonde	75 (22%)	10 (29%)	2 (13%)
Scant	19 (6%)	4 (11%)	0 (0%)
Red	7 (2%)	0 (0%)	0 (0%)
Mechanism of injury			
Fall	179 (52%)	16 (44%)	7 (44%)
Sports	39 (11%)	4 (11%)	3 (19%)
Bicycle	24 (7%)	6 (17%)	2 (13%)
Motor vehicle crash	21 (6%)	2 (6%)	1 (6%)
Assault/NAT	18 (5%)	1 (3%)	0 (0%)
Pedestrian struck	16 (5%)	2 (6%)	0 (0%)
Hit with blunt object	10 (3%)	0 (0%)	0 (0%)
Motorcycle	3 (1%)	0 (0%)	0 (0%)
Other	34 (10%)	5 (14%)	3 (19%)
Intubated	6 (2%)	3 (8%)	2 (13%)
Disposition			
Home	251 (73%)	5 (14%)	1 (6%)
Floor	74 (22%)	18 (50%)	6 (38%)
PICU	19 (6%)	13 (36%)	9 (56%)
Glasgow Coma Scale, Median [IQR]	15.0 [15, 15] Range 7–15	15.0 [15, 15.0] Range 7–15	15.0 [14, 15] Range 9–15

* Number of subjects used for race (336, 33, 14) and ethnicity (322, 32, 13) calculations for evaluable patients, patients with any hematoma and patients with a hematoma within the detection limit of the Infrascanner, respectively due to missing data.

*SD*, standard deviation; *NAT*, non-accidental trauma; *PICU*, pediatric intensive care unit; *IQR*, interquartile range.

**Table 2:** Infrascanner diagnostic performance.

	Any hematoma (95% CI)	Hematomas within the detection limit of the infrascanner (95% CI)
Negative predictive value	93% (209/224) (89% – 96%)	99% (221/224) (96% – 100%)
Positive predictive value	18% (21/120) (11% – 26%)	11% (13/120) (6% – 18%)
Sensitivity	58% (21/36) (41% – 75%)	81% (13/16) (54% – 96%)
Specificity	68% (209/308) (62% – 73%)	67% (221/328) (62% – 72%)
Predictive Youden’s index	0.11 (0.10 – 0.15)	0.09 (0.08 – 0.12)
Youden’s index	0.26 (0.27 – 0.32)	0.49 (0.47 – 0.51)

*CI*, confidence interval.

**Table 3:** Clinically important traumatic brain injury outcomes and detection limit of the handheld scannner.

	Hematoma within the detection limit of the infrascanner		Hematoma outside the detection limit of the infrascanner	

	Infrascanner Positive (N = 13)	Infrascanner Negative (N = 3)	Infrascanner Positive (N = 8)	Infrascanner Negative (N = 12)
Clinically important TBI	10 (76.9%)	2 (66.7%)	3 (37.5%)	2 (16.7%)
Death	--	--	--	--
Neurosurgical intervention	4 (30.8%)	--	--	--
Intubation ≥ 24 hours	--	--	--	--
Hospital admission ≥ 2 nights	10 (76.9%)	2 (66.7%)	3 (37.5%)	2 (16.7%)

*TBI*, traumatic brain injury.
