# Supplementary figures and images for: Intracranial Traumatic Hematoma Detection in Children Using a Portable Near-infrared Spectroscopy Device
Source: West J Emerg Med. 2021 Mar 24;22(3):782–91. doi: 10.5811/westjem.2020.11.47251 (PMC8203002; doi:10.5811/westjem.2020.11.47251)

Supplemental Figure 1: Specificity for any hematoma by number of scans performed per operator

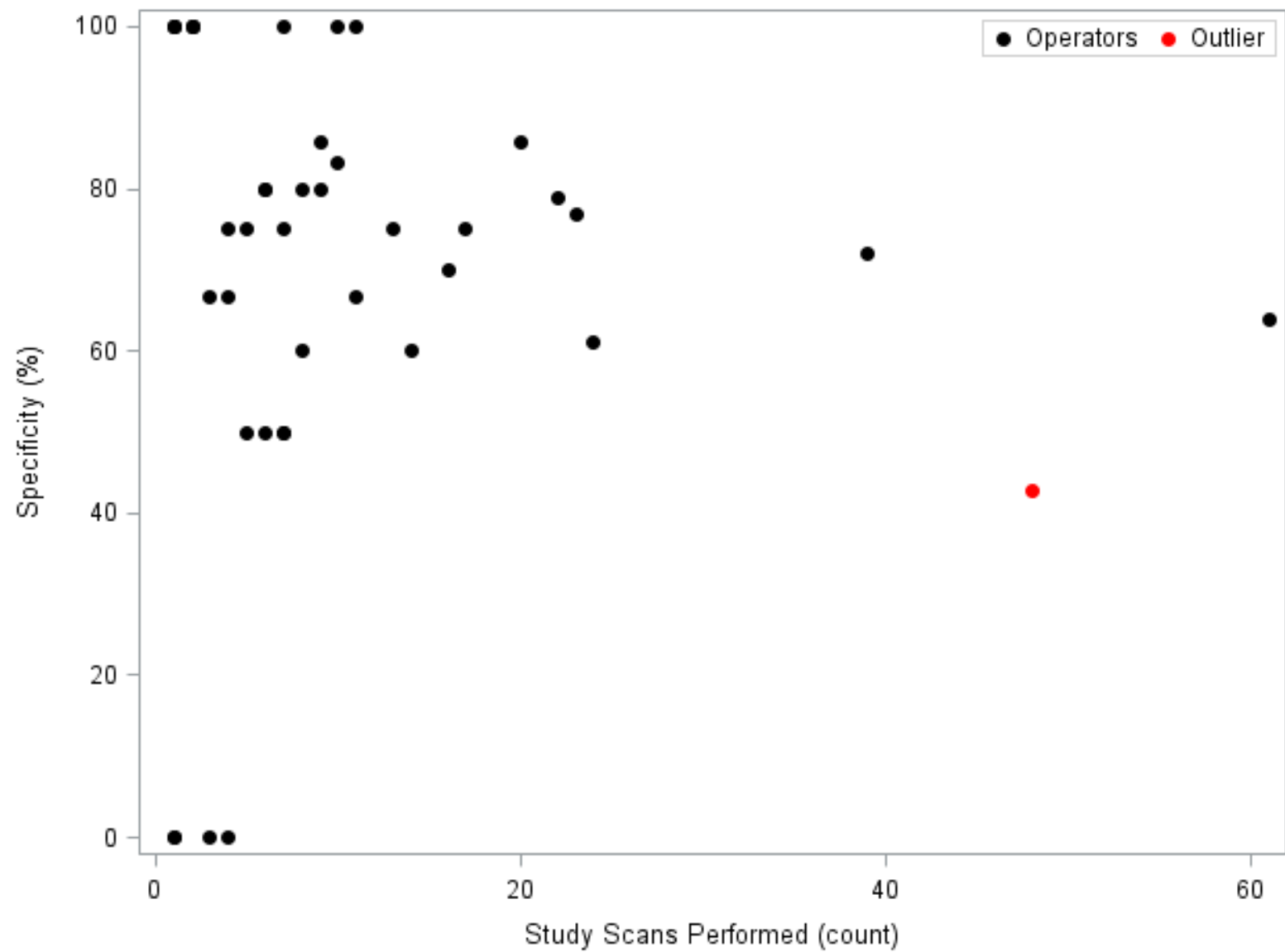

Supplement: Supplementary file 1 [file wjem-22-782-s001.pdf]
