# Supplementary material for: Intracranial Traumatic Hematoma Detection in Children Using a Portable Near-infrared Spectroscopy Device
Source: West J Emerg Med. 2021 Mar 24;22(3):782–91. doi: 10.5811/westjem.2020.11.47251 (PMC8203002; doi:10.5811/westjem.2020.11.47251)
Supplement: Supplementary file 2 [file wjem-22-782-s002.pdf]

Supplemental Table 1: Infrascanner diagnostic performance for subgroups

Subgroup	Sensitivity	Specificity	Negative Predictive Value	Positive Predictive Value
**Overall**	81% (13/16)<br>54%, 96%	68% (209/308)<br>63%, 73%	99% (221/224)<br>96%, 100%	11% (13/120)<br>6%, 18%
**Age**				
Quartile 1<br>(0 to 4.97 Years)	100% (5/5)<br>48%, 100%	68% (50/74)<br>56%, 78%	100% (55/55)<br>94%, 100%	16% (5/31)<br>5%, 34%
Quartile 2<br>(4.98 to 9.53 Years)	50% (1/2)<br>1%, 99%	67% (54/81)<br>55%, 77%	98% (57/58)<br>91%, 100%	4% (1/28)<br>0%, 18%
Quartile 3<br>(9.54 to 13.77 Years)	71% (5/7)<br>29%, 96%	75% (55/73)<br>64%, 85%	97% (56/58)<br>88%, 100%	18% (5/28)<br>6%, 37%
Quartile 4<br>(> 13.77 Years)	100% (2/2)<br>16%, 100%	63% (50/80)<br>51%, 73%	100% (53/53)<br>93%, 100%	6% (2/33)<br>1%, 20%
p-value	0.34	0.39	0.32	0.20
**Race**				
Asian	-	53% (9/17)<br>28%, 77%	100% (10/10)<br>69%, 100%	-
Black /African American	100% (2/2)<br>16%, 100%	70% (81/115)<br>61%, 79%	100% (81/81)<br>96%, 100%	5% (2/39)<br>1%, 17%
White	89% (8/9)<br>52%, 100%	66% (97/146)<br>58%, 74%	99% (105/106)<br>95%, 100%	13% (8/62)<br>6%, 24%
Other	100% (3/3)<br>29%, 100%	72% (18/25)<br>51%, 88%	100% (20/20)<br>83%, 100%	30% (3/10)<br>7%, 65%
p-value	0.74	0.49	0.79	0.10
**Skin Color**				
Black/Olive/Brown	67% (4/6)<br>22%, 96%	67% (114/171)<br>59%, 74%	98% (116/118)<br>94%, 100%	6% (4/65)<br>2%, 15%
Light/White	90% (9/10)<br>56%, 100%	69% (95/137)<br>61%, 77%	99% (105/106)<br>95%, 100%	16% (9/55)<br>8%, 29%
p-value	0.25	0.62	0.63	0.07
**Hair Color**				
Black/Brown	77% (10/13)<br>46%, 95%	67% (148/221)<br>60%, 73%	98% (151/154)<br>94%, 100%	11% (10/88)<br>6%, 20%
Blond/Red/Scant	100% (2/2)<br>16%, 100%	70% (61/87)<br>59%, 79%	100.0% (70/70)<br>95%, 100%	6% (2/31)<br>1%, 21%
p-value	0.45	0.59	0.24	0.43
**Site**				
Children’s Hospital of Philadelphia	86% (6/7)<br>42%, 100%	70% (121/173)<br>63%, 77%	99% (125/126)<br>96%, 100%	10% (6/60)<br>4%, 21%
Boston Children’s Hospital	75% (3/4)<br>19%, 99%	66% (37/56)<br>52%, 78%	98% (41/42)<br>87%, 100%	13% (3/24)<br>3%, 32%
Children’s Hospital Colorado	80% (4/5)<br>28%, 99%	65% (51/79)<br>53%, 75%	98% (55/56)<br>90%, 100%	11% (4/36)<br>3%, 26%
p-value	0.91	0.66	0.70	0.94

Subgroup	Sensitivity	Specificity	Negative Predictive Value	Positive Predictive Value
**Number of Lobes Measured**				
3	78% (7/9)<br>40%, 97%	69% (90/130)<br>61%, 77%	98% (95/97)<br>93%, 100%	13% (7/52)<br>6%, 26%
4	86% (6/7)<br>42%, 100%	67% (119/178)<br>59%, 74%	99% (126/127)<br>96%, 100%	9% (6/68)<br>3%, 18%
p-value	0.69	0.66	0.41	0.42
**Device Mode**				
Independent	85% (11/13)<br>55%, 98%	71% (145/205)<br>64%, 77%	99% (155/157)<br>95%, 100%	14% (11/76)<br>7%, 24%
Guided	67% (2/3)<br>9%, 99%	62% (64/103)<br>52%, 72%	99% (66/67)<br>92%, 100%	5% (2/44)<br>1%, 15%
Chi-Square p-value	0.47	0.13	0.90	0.09
**Hematoma Location**				
Epidural	67% (6/9)<br>30%, 93%	-	-	-
Subdural	100% (4/4)<br>40%, 100%	-	-	-
Intracerebral	100% (3/3)<br>29%, 100%	-	-	-
p-value	0.24	-	-	-
**Hematoma volume**				
>3.5-13.0 mL	88% (7/8)<br>47%, 100%	-	-	-
>13.0 mL	75% (6/8)<br>35%, 97%	-	-	-
p-value	0.52	-	-	-
**Scalp Hematoma**				
Yes	80% (4/5)<br>28%, 99%	66% (60/91)<br>55%, 76%	99% (68/69)<br>92%, 100%	11% (4/38)<br>3%, 25%
No	82% (9/11)<br>48%, 98%	69% (149/217)<br>62%, 75%	99% (153/155)<br>95%, 100%	11% (9/82)<br>5%, 20%
p-value	0.93	0.64	0.92	0.94

Results presented as proportion (numerator/denominator), lower, upper 95% confidence interval. Sensitivity, negative predictive value, and positive predictive value are based on evaluable intracranial hematomas within the detection limit of the device. Specificity is based on absence of any hematoma regardless of depth and size to prevent penalizing successful detection of smaller and deeper hematomas. A two-sided p-value from a chi-square test comparing the estimates between subgroups is reported. Only sensitivity is reported for hematoma location and volume, since these factors are not applicable to patients without hematomas.
